# Herpes Vegetans: An Atypical Presentation of Herpes Simplex Virus (HSV) in AIDS

**DOI:** 10.7759/cureus.93832

**Published:** 2025-10-04

**Authors:** Gregory R Alfieri, Elizabeth Audino, Amaury Diaz

**Affiliations:** 1 Medicine, Nova Southeastern University Dr. Kiran C. Patel College of Osteopathic Medicine, Fort Lauderdale, USA; 2 Infectious Disease, Polk County Health Department, Bartow, USA

**Keywords:** atypical herpes simplex virus, herpes simplex, herpes vegetans, hiv/aids, immunocompromised patients, valacyclovir treatment

## Abstract

Herpes vegetans (HV) is an atypical presentation of herpes simplex virus (HSV) that occurs in immunocompromised patients, specifically those with human immunodeficiency virus (HIV) infection or individuals diagnosed with acquired immunodeficiency syndrome (AIDS). Lesions typically appear as verrucous, hypertrophic masses in the inguinal and anogenital region.

We present a case of a male patient with AIDS and a history of noncompliance to anti-retroviral therapy who developed large, verrucous HV lesions on both the penis and inguinal regions. Following imaging and work-up to rule out a suspected malignancy, a biopsy was performed, confirming HSV etiology. Subsequent medical therapy with valacyclovir 1000 mg daily for two months resulted in complete resolution of all lesions.

Due to the atypical appearance of HV lesions, diagnostic workup for malignancy without HSV testing is often prompted, which effectively delays the timeline from lesion onset to therapy induction. This delay can result in increased morbidity for patients, highlighting the necessity for increased awareness regarding HV in immunocompromised patients. Although cases of valacyclovir resistance have been reported, it still remains the first-line therapy.

Although HV is rare and its appearance can often mimic malignancy, a prompt biopsy with HSV staining is warranted in new atypical lesions in an immunocompromised patient. A timely diagnosis can increase the chance of full resolution, without the need for more invasive therapeutic approaches.

## Introduction

Herpes simplex virus (HSV) is an enveloped DNA virus within the Herpesviridae family. Although HSV 1 is classically defined by orofacial infections and HSV 2 as genital herpes, the clinical presentations can vary widely. These presentations range from asymptomatic infection to mucocutaneous conditions, such as orolabial and ocular infection. Herpes vegetans (HV) is an uncommon HSV infection seen in patients who are immunocompromised, such as those with human immunodeficiency virus (HIV) or acquired immunodeficiency syndrome (AIDS) with very low CD4+ lymphocyte cell counts (<50 cells/mm³) [1,2]. HIV is a virus that weakens the immune system by attacking white blood cells, specifically CD4+ lymphocytes, and AIDS is the most advanced disease stage, defined by CD4+ cell counts less than 200 cells/mm³ [3]. In patients with AIDS, HV can be clinically described as exophytic exudative ulcers and papillomatous vegetations in the genital and inguinal regions [2]. Diagnosis and treatment in this patient demographic prove to be challenging, as the lesion commonly imitates malignancy and is often resistant to acyclovir [1,2]. We present a case of multiple large, atypical HV lesions in a patient with AIDS.

## Case presentation

**Figure 1:**
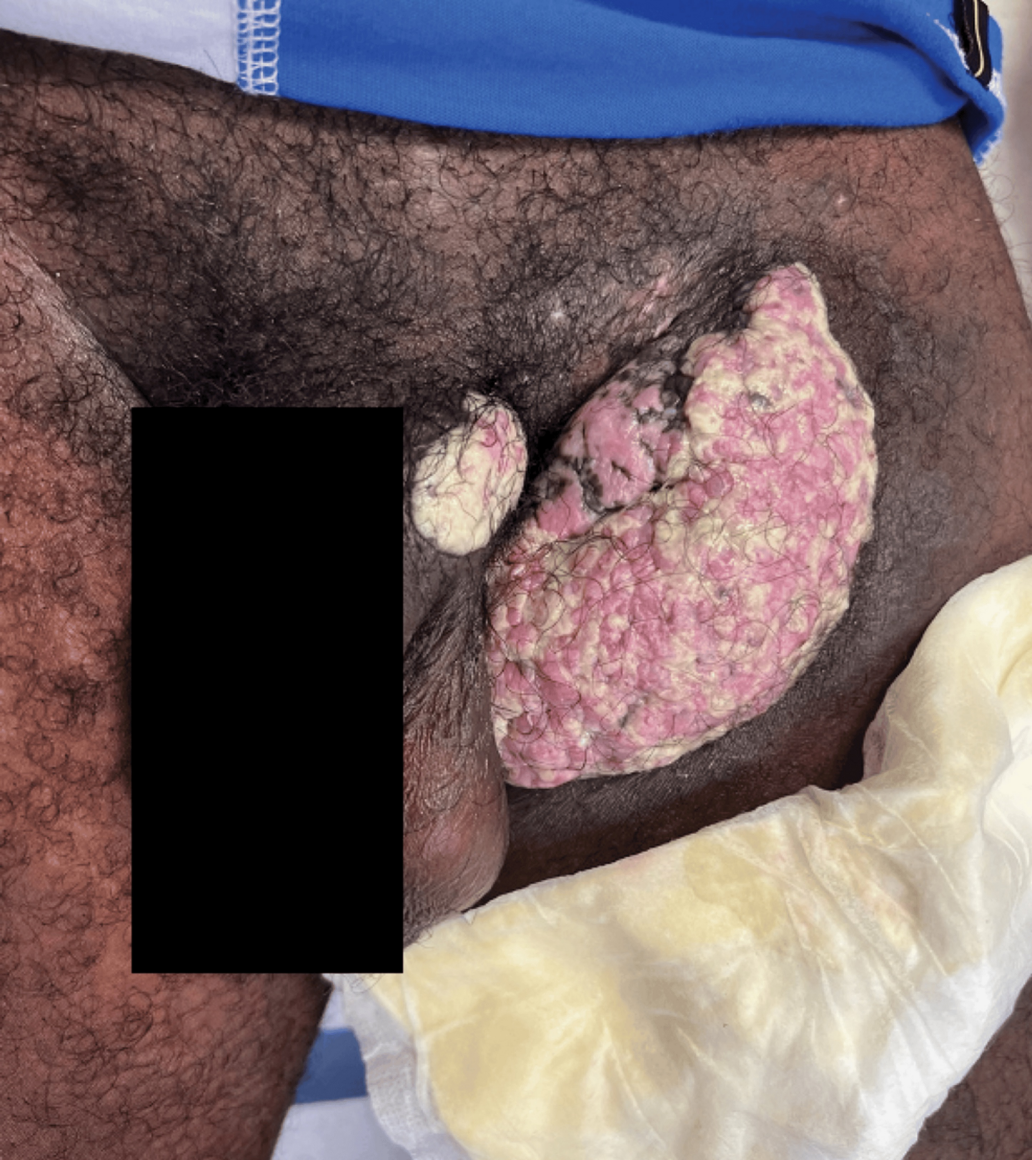
Penile base and inguinal HV lesions HV: Herpes vegetans

**Figure 2:**
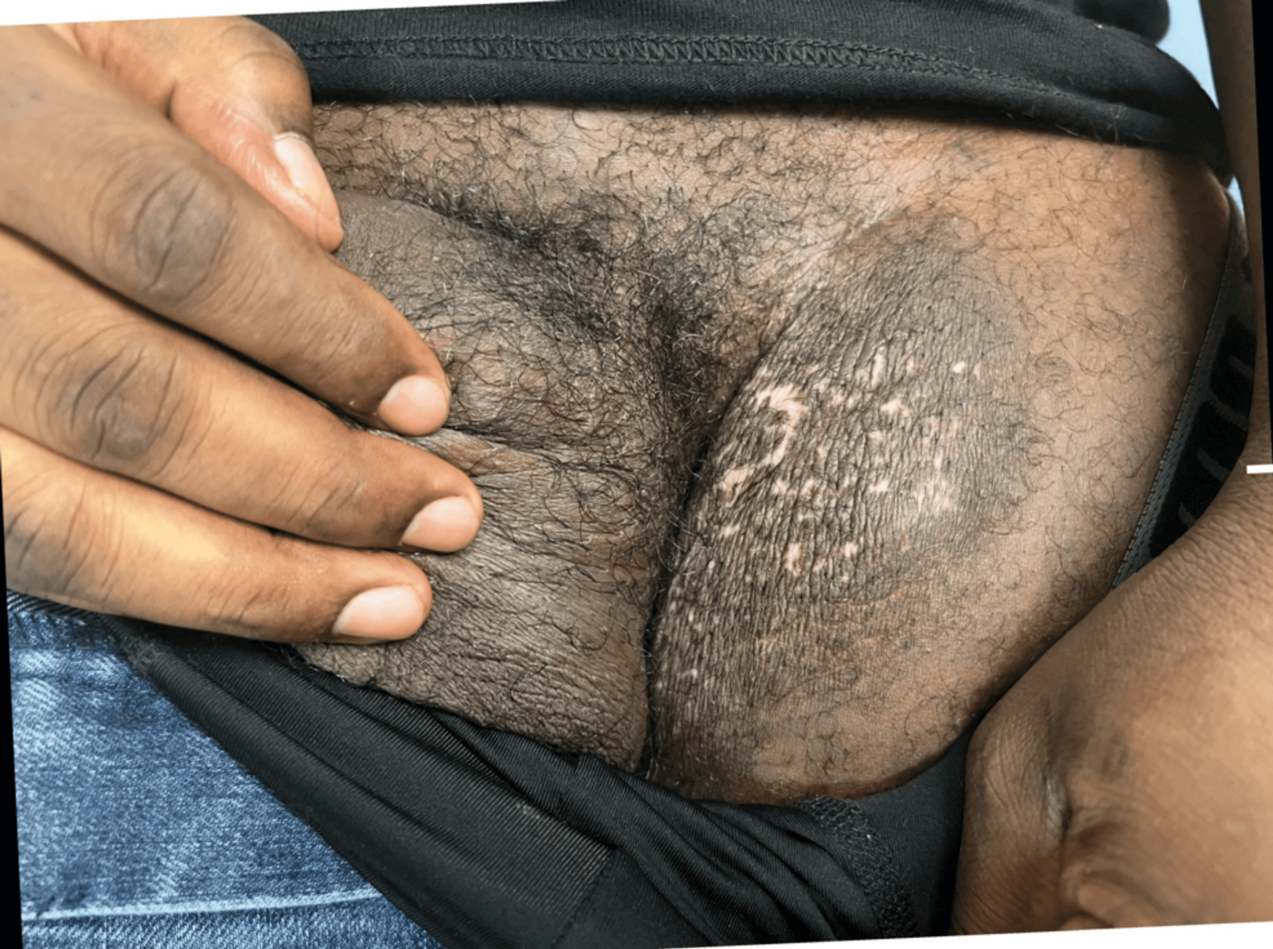
Resolution of HV lesions HV: Herpes vegetans

A 32-year-old African American man presented for evaluation of a mass at the left inguinal and penile base, which he reported had been present for over a year. His medical history was significant for HIV/AIDS, which was diagnosed eight years prior, as well as a history of non-compliance to antiretroviral therapy (ART). One month prior, the patient was hospitalized for the biopsy of a potential malignancy, with suspicion for squamous cell carcinoma and giant condylomata acuminata of Bushke-Löwenstein (GCBL), a slow-growing growing verrucous, or wart-like, tumor of the penis and anorectal region. A computed tomography (CT) scan of the pelvis with contrast was performed at the time of hospitalization and showed irregular skin thickening overlying the left inguinal area with extension to the left base of the scrotum and uppermost anterior medial left thigh. Physical exam revealed a macerated, verrucous left inguinal mass with serous malodorous drainage. A second mass of a smaller size was also observed at the left upper aspect of the penile base (Figure 1). The CD4+ lymphocyte count at the time of physical exam was 21 cells/mm^3^. An additional biopsy was performed, with the intent of future excision. Further studies included a tissue Gram stain, acid-fast bacilli (AFB) stain, and cultures for HSV, AFB, bacteria, and fungi. Upon receipt of the pathology report, the lesion was positive for HSV DNA, with the report describing abundant inflammatory debris, eosinophilic microabscesses, and prominent HSV cytopathic effects. At this time, excision was no longer considered, and the patient was treated with valacyclovir 1000 mg twice a day orally for two months. To date, the lesions have resolved (Figure 2), and valacyclovir was tolerated without adverse reactions.

## Discussion

Atypical HV lesions, most commonly found in the anogenital region, have appeared in patients with a history of chronic immunodeficiency and AIDS [4,5]. HV is a rare manifestation of HSV infection seen in immunocompromised patients, manifesting as verrucous, proliferative, or ulcerative lesions, with a characteristic chronic, persistent course [6,7]. The presentation of HV contrasts with typical HSV lesions in immunocompetent patients, as characteristic tender, clustered vesicles are typically absent [7]. Although the exact pathophysiology of HV remains unclear [5], an important documented interaction between HIV and HSV-2 is increased mucosal shedding of HSV-2 in patients with HIV compared to those without HIV. This may result in more frequent and severe anogenital HSV infections [8,9], which may not respond to therapy, thus progressing to chronic severe anogenital infections [8,9]. The majority of these patients have CD4+ counts lower than 500 cells/mm^3^ and atypical presentations include HV and hypertrophic pseudo-tumors mimicking malignancies [8]. Another proposed mechanism is that prolonged cutaneous inflammation leads to hyperproliferation of keratinocytes through cytokine secretion, which can lead to atypical presentations of HSV [10]. 

The clinical features of HV often promote suspicion of other conditions, including malignancy, condyloma acuminatum, and deep fungal and mycobacterial infections [1,2]. In our case, suspicion of GCBL prompted inpatient imaging and biopsy, yet ideally, HSV staining using repeated biopsy tissue samples should be performed with tissue culture as confirmation [2,5,7,11]. Interestingly, superficial viral cultures of nodules are often negative [12]. HSV staining was not performed in the emergency room and was completed at a later date at our clinic, further delaying proper diagnosis and treatment. This prompts the necessity for education of infectious disease and HIV specialists regarding timely identification of HV presentation.

Although cases of ineffective treatment of HV with valacyclovir have been reported [4], valacyclovir is largely effective and remains a first-line agent for the treatment of HV lesions [5]. Widespread resistance to acyclovir has been reported, in which case successful treatment options include foscarnet, cidofovir, trifluridine, and imiquimod [5,11,12]. Compared with acyclovir, valacyclovir is both more bioavailable and effective in the resolution of cutaneous HSV infections [13]. Surgical resection should also be considered in immunocompromised patients with chronic severe anogenital herpes simplex infections who fail to respond to antiviral therapy, as it has been previously reported that excision followed by acyclovir or valacyclovir can prevent lesion recurrence [8,13]. It is also recommended that antiviral therapy be administered for a longer period of time for HV lesions and continued orally until clearance of all lesions occurs [7].

Due to the rarity of the condition, it is vital for physicians to be aware of the presenting signs of HV so that diagnostic work-up can begin swiftly. Providers should know that the presence of HV is strongly suggestive of an underlying associated immunodeficiency, particularly HIV/AIDS [5]. Timely diagnosis allows prompt initiation of ART, reducing the mortality and morbidity associated with unmanaged HIV/AIDS. In addition, although proposed theories exist, further research is needed to determine the pathophysiology of HV, as it remains unknown [5]. If the mechanism can be explained, development of better treatment and prophylactic regimens can begin, as some evidence exists regarding increased survival of patients with AIDS and previous exposure to HSV with chronic use of suppressive therapy with acyclovir [13].

## Conclusions

Our case highlights a patient with AIDS who initially received a CT and biopsy as malignancy was highly suspected. Upon proper diagnosis, valacyclovir treatment was successful, further confirming the efficacy of this pharmacological option. The low prevalence of this disease and lack of awareness may contribute to the fact that HV may not be thought of as a differential diagnosis. Furthermore, presentation of HV should prompt further diagnostic testing for suspicion of immunosuppressed states, such as HIV.

## References

[1] Sirait SP, Indriatmi W (2022). Herpes vegetans, an atypical herpes lesion in HIV patient: a case report. Dermatol Reports.

[2] Helmandollar K, DiStefano N, Moy J (2023). Penile herpes vegetans in a patient with well-controlled HIV. Cutis.

[3] (2025). HIV and AIDS. https://www.who.int/news-room/fact-sheets/detail/hiv-aids.

[4] Frey C, Dinkins J, Suah S, Merkel K (2023). Herpetic pseudotumor of the nostril: a report of facial herpes vegetans in a patient with chronic lymphocytic leukemia. Cureus.

[5] Patel AB, Rosen T (2008). Herpes vegetans as a sign of HIV infection. Dermatol Online J.

[6] Liu MJ, Li J (2023). Herpes vegetans. N Engl J Med.

[7] Koushk-Jalali B, Oellig F, Wieland U, Kreuter A (2019). Herpes simplex vegetans in a patient with primary myelofibrosis. Infection.

[8] Arinze F, Shaver A, Raffanti S (2017). Surgical excision for recurrent herpes simplex virus 2 (HSV-2) anogenital infection in a patient with human immunodeficiency virus (HIV). Infection.

[9] Strick LB, Wald A, Celum C (2006). Management of herpes simplex virus type 2 infection in HIV type 1-infected persons. Clin Infect Dis.

[10] Cury K, Valin N, Gozlan J (2010). Bipolar hypertrophic herpes: an unusual presentation of acyclovir-resistant herpes simplex type 2 in a HIV-infected patient. Sex Transm Dis.

[11] Hagiwara H, Iwata Y, Saito K (2018). Herpes vegetans accompanied by Good's syndrome. J Dermatol.

[12] Ronkainen SD, Rothenberger M (2018). Herpes vegetans: an unusual and acyclovir-resistant form of HSV. J Gen Intern Med.

[13] Simonsen M, Nahas SC, Silva Filho EV, Araújo SE, Kiss DR, Nahas CS (2008). Atypical perianal herpes simplex infection in HIV-positive patients. Clinics (Sao Paulo).

